# Barriers to Emergency Obstetric Care Services in Perinatal Deaths in Rural Gambia: A Qualitative In-Depth Interview Study

**DOI:** 10.5402/2011/981096

**Published:** 2011-06-30

**Authors:** Abdou Jammeh, Johanne Sundby, Siri Vangen

**Affiliations:** ^1^Section for International Health, Department of General Practice and Community of Medicine, Institute of Health and Society, University of Oslo, P.O. BOX 1130, Blindern, 0318 Oslo, Norway; ^2^Reproductive and Child Health Programme, Ministry of State for Health and Social Welfare, Banjul, Gambia; ^3^National Resource Centre for Women's Health, Department of Obstetrics and Gynaecology, Oslo University Hospital, Postboks 4950, 0424 Oslo, Norway

## Abstract

*Objective*. The Gambia has one of the world's highest perinatal mortality rates. We explored barriers of timely access to emergency obstetric care services resulting in perinatal deaths and in survivors of severe obstetric complications in rural Gambia. *Method*. We applied the “three delays” model as a framework for assessing contributing factors to perinatal deaths and obstetric complications. Qualitative in-depth interviews were conducted with 20 survivors of severe obstetric complications at home settings within three to four weeks after hospital discharge. Family members and traditional birth attendants were also interviewed. The interviews were translated into English and transcribed verbatim. We used content analysis to identify barriers of care. *Results*. Transport/cost-related delays are the major contributors of perinatal deaths in this study. A delay in recognising danger signs of pregnancy/labour or decision to seek care outside the home was the second important contributor of perinatal deaths. Decision to seek care may be timely, but impaired access precluded utilization of EmOC services. Obtaining blood for transfusion was also identified as a deterrent to appropriate care. *Conclusion*. Delays in accessing EmOC are critical in perinatal deaths. Thus, timely availability of emergency transport services and prompt decision-making are warranted for improved perinatal outcomes in rural Gambia.

## 1. Introduction

The Millennium Development Goal (MDG) 4 of the United Nations, to reduce under-five mortality by two thirds, and MDG 5, to reduce maternal mortality by three-quarters between 1990 and 2015, are closely related [1]. Every year, 4 million newborn babies die in the first month of life. Of these, 99% occur in low- and middle-income countries [2]. Maternal and perinatal mortality rates are still alarmingly high, especially in sub-Saharan Africa, where little progress has been made over recent decades [3]. More than 515,000 women die as a result of pregnancy-related complications each year, of which half are in Africa [4]. In addition, it is estimated that 880,000 babies are stillborn in sub-Saharan Africa, a fact which remains invisible in the policy agenda [5]. However, the majority of these newborn and maternal deaths are due to preventable causes [2]. Undoubtedly, maternal interventions do benefit children, particularly in relation to newborn survival [6], but preventing deaths in newborn babies has not been a major focus of child survival or safe motherhood programmes, particularly in Africa [2]. Deaths due to childbearing are not only personal tragedies, but can be ruinous to the family, especially to children and the community.

Access to appropriate maternity care including prompt referrals to emergency obstetric care (EmOC) services and skilled birth attendance could significantly reduce both perinatal and maternal mortality and/or morbidity [4, 7]. However, women in many countries in sub-Saharan Africa continue to have restricted access to skilled birth attendants [8]. While more than half of all births in sub-Saharan Africa occur without the presence of a skilled attendant, nearly all births in developed nations take place with the assistance of a skilled birth attendant [8, 9].

In many West African countries, maternal and neonatal mortality is highest in rural areas where access to EmOC service is inhibited by vast geographic distances to health facilities and scarce resources [10]. Despite the efforts by the Gambian Government in adopting the primary health care (PHC) strategy, the aim of which was to make health care more accessible and affordable to the majority of Gambians, physical access to health services has been hampered by a rapidly growing population, inadequate financial and logistic support, gross shortage of skilled human resource for health, high staff attrition, and an inefficient referral mechanism [11]. Poverty and ignorance have, in some instances, led to inappropriate health seeking delivery behaviour and contributed to ill health [11]. Although the percentage of population living within 5 km of a primary health care facility has improved over the years, the availability, accessibility, and quality of EmOC services in The Gambia are below required standards [12]. While the number of comprehensive EmOC facilities per 500,000 populations is within the recommended level, the number of basic EmOC facilities is far below the United Nations recommended level [13]. Hence, a large number of women in remote settings still continue to travel some distance to reach a basic EmOC facility or resort to home deliveries. It should therefore be noted that nearly two-thirds of all births in Gambia take place in home settings, mostly attended to by traditional birth attendants (TBAs) and/or family members [13]. Thus, most of the deaths and disabilities that women face in these settings could have been averted if the services of a skilled birth attendant were available and accessible to those women in need. However, The Gambia has yet to formulate a policy on skilled attendance at birth.

The purpose of this study was to provide a better understanding of the barriers to timely access to emergency obstetric care in perinatal deaths in a rural setting in The Gambia.

## 2. Model

The fundamental constructs in understanding the causes of maternal deaths is the “three delays” model developed by Thaddeus and Maine [14]. These are (1) delay in recognising danger signs/decision to seek care, (2) delays in reaching a medical facility, and (3) delay in receiving appropriate care once a facility is reached. We adopted the “three delays” model as a framework in assessing the barriers to obstetric care in survivors of severe obstetric complications. Twenty survivors with stillbirth or perinatal death were question. The “three delays” model provides a valuable theoretical account in examining composite interactions between women's ability to recognise danger signs during pregnancy and child birth, the problems they encounter in reaching an EmOC facility and the quality of care received at the facility. The model identifies key time periods in pregnancy and childbirth during which delays can occur that have direct consequences on both maternal and neonatal survival. Because perinatal and maternal morbidity and mortality share many risk factors, the “three delays” model may assist in perinatal health outcome assessment. The model has been applied previously in analyses of newborn death in Tanzania and Uganda [15, 16]. 

## 3. Material and Methods

### 3.1. Study Setting/Area

The study was carried in Bansang hospital located on the south bank of The Gambia in the Central River Region (CRR). The CRR has an under-five mortality of rate of 165/1000, the highest in the country [11]. Bansang hospital is the oldest rural hospital and serves the eastern part of the country including the catchment areas of Upper River Region (URR), covering about one-third of the country's population, mainly subsistence farmers. The hospital, which was purposively selected for this study serves as a referral point for nearly sixteen peripheral health centres that provide first-level care to the inhabitants of CRR and URR. Basic and comprehensive EmOC service is not available at any of these health centres except one, which is not fully operational. Thus, Bansang hospital is the only facility that provides comprehensive EmOC services in this part of the country. Though the catchment area population of this comprehensive EmOC facility is below the 500,000 minimum outlined in the UN guidelines, reaching this hospital still remains a challenge for remote rural dwellers. Poor road conditions and/or transportation networks and river crossings are practical factors that compromise access and utilisation of emergency obstetric care services. Most of the maternity admissions and deliveries in this hospital are high risk and the bulk of the referrals are during labour. However, there are no Gambian medical doctors or gynaecologists in this hospital and as such they depend to a large extent on the expatriate doctors on technical missions. Since all the specialist doctors are non-Gambian, the withdrawal of such assistance could affect the quality of obstetric care services in this hospital. The institutional fresh stillbirth rate was calculated at 49.6 per 1000 births [17]. In addition to operating as a referral hospital, it also provides outpatient services as well as static and mobile reproductive and child health services. Antenatal care is provided by nurses and or midwives within here and on outreach clinic.

### 3.2. Study Population/Design

Individual in-depth interviews were carried out with women who sought obstetric care and delivered in Bansang hospital a month prior to data collection. The study population included surviving women with the following diagnostic criteria antepartum haemorrhage (placenta previa/abruption placenta *n* = 9), pregnancy-induced hypertension (pre-eclampsia/eclampsia *n* = 4), prolonged and/or obstructed labour (*n* = 5), and severe anaemia (*n* = 2). Key informants (family member(s) and traditional birth attendants, TBAs) were also interviewed. A total of 20 women who delivered between April 2010 and June 2010 were purposively selected based on the characteristics similar to women from a preceding study [17], resource availability, and geographic location. All of them had a stillbirth (*n* = 19) or neonatal death (*n* = 1). Prior to discharge from the hospital, individual consent for participation in the study was obtained from the women. In addition, telephone numbers and permanent addresses were also provided to facilitate the data collection process. The principal investigator (AJ), assisted by experienced community health workers, visited the selected participants in their homes and administered semistructured interview guides in the local language they understood in the presence of relative (s) and TBAs who also acted as key informants. 

The interviews concentrated on the following respondents' ability in recognising obstetric complications, care seeking/decision making procedures, and the processes of getting care from home to the hospital. The interviews were translated into English and transcribed verbatim using content analysis [18]. The main topics identified were examined for similarities and phrases that represented similar topics or ideas were further clustered together in categories. Furthermore, typical statements were used for citation. Data analysis was done manually and continuously during the data collection period. Informed consent was gained from all respondents before commencement of interviews. This entailed an explanation of the purpose of the study, a guarantee of confidentiality relating to the information to be given and assurance that participation would not have any negative bearing on the availability and provision of health care to them or their families. The ethics committee of Norway and the Joint Gambia Government/Medical Research Council Review board approved the study.

## 4. Results

### 4.1. Characteristics of Women

The mean age of the women was 29 years, with a range of 18–40 years. The mean parity was 4, and all except one attended antenatal care. None of the women ever attended formal school and all were married except one. All the twenty women delivered at the study site but only three sought initial care at another health centre. Of the twenty women, 19 had a stillbirth while one had an early neonatal death before discharge from the hospital. The first delays occurred in 30% of the cases, second delays in 50%, and the third delays occurred in 20% of the cases (Table 1).

### 4.2. Recognising Danger Signs during Pregnancy and Labour

Awareness and/or recognition of an obstetric complication among pregnant women and within their communities is the first step to initiating appropriate and timely referrals to essential obstetric and newborn care, hence reducing the time needed to make a decision to seek care, which is called the “first delay.” However, most women and families in developing countries, particularly in rural settings seldom know in detail the danger signs of pregnancy and childbirth, or they misinterpret or “hope for the best.” As a result many do not seek health care because they do not know that they have a problem. A severely anaemic woman narrated her ordeal:

*“*…*When I was pregnant I usually feel tired especially during late evenings*…*I have had sleepless nights all the time*…*previous pregnancies I was always strong but this time I am weak and can't do anything*…*I fell down and hurt my legs but was taken to my mothers' house to rest.” *

Giving her version of the above scenario the mother-in-law commented:

*“In the village women are responsible for all household chores*…*I am sure the tiredness my daughter-in-law got when she was pregnant is due to tedious domestic work*…*she work *[*sic*] *from morning to evening without rest*…*And *…*this is not good for a pregnant woman.” *

An informed individual is better placed to make reasonable decisions in emergency situations. At times women reflect on experiences during their last pregnancy and try to apply this to circumstances surrounding them. A 29-year-old woman who had antepartum haemorrhage narrated:

*“Before labour started*…*I had a tensed abdomen but there was no pain and the baby is not moving as usual*…*I have no idea what the problem was*…*I have never given birth at a health centre or hospital so I thought everything was going to be okay.” *

However, some participants were well informed and aware of an imminent danger threatening the life of the pregnant woman. Persuading and getting the consent of other family members to seek help outside of the home is sometimes not as difficult as one might think. Narratives of a sister-in-law to a 32-year-old woman with obstructed labour and history of previous caesarean section (C/S) supported this:

*“The problem started at home when she was in labour*…*I recognized that she was in pain for a very long time with no improvement*…*Her (referring to the woman) abdomen was very big and tensed*…*and I feared that she might not be able to deliver on her own again*…*she have *[*sic*]* had two previous operations (C/S) so I thought she would not be able to deliver on her own again*…*Her abdomen was too big*…*the pain was increasing yet there was no sign of delivery and everybody was worried*…*So I suggested that we go to the hospital so that they could assist to deliver her through operation (C/S) once more*…*The traditional birth attendant was present and everybody agreed.” *

### 4.3. Socio-Cultural Belief/Family Decision-Making

Social factors tend to influence women's likelihood to seek health care during pregnancy and labour. In rural areas of The Gambia the decision to seek early delivery at a health centre and/or hospital is usually influenced by mother-in-laws. Visiting the hospital early in labour was viewed by most mother-in-laws as unnecessary; they would prefer to prepare traditional herbs while waiting for established labour. This they believed will make the pelvic bones flexible thus facilitating quick and safe delivery even without going to the hospital. A 29-year-old woman with obstructed labour narrated her encounter with the mother-in-law:

“*My mother-in-law argued that I should not report to the hospital when I started having abdominal pains*…*that*…*it was too early to go and as a result we may stay at the hospital for a long time before I deliver*…*she decided to cook traditional herbs for me while waiting for established labour*…*she believed the herbs would be useful in making the delivery quick and less painful.” *

Even in situations where awareness of danger signs during pregnancy and labour helps women and families to recognize a complication, this does not automatically translate into a prompt decision to seek help outside of the home. How severe they consider the problem is equally an important determinant of deciding when to act. Mother of an 18-year-old single eclamptic woman explained her ordeal:

*“She got pregnant before the traditional married ceremony was done*…*she hide *[*sic*]* the pregnancy and never attended antenatal care. She developed swollen legs latter on*…*and started complaining of severe headache...and heart burn*…*she was not feeling well the day she went into labour since the early hours of the morning*…*but I felt it was too early to go to the hospital so I prepared local medicine for her. We remained home until very late in the evening when she started fitting*…*and the baby died in the womb.” *

This is another testimony from a husband of woman with antepartum haemorrhage:

*“*…*She (referring to his wife) started bleeding in the night*…*but we thought everything will be all right even though I know that bleeding in pregnancy is dangerous*…*while waiting for sunrise*…*We gave her holly *[sic]* water*…*but this does *[sic]* not help. After the local remedies failed*…*we thought the best thing to do was to take her to the hospital*…*”. *

### 4.4. Transportation, Distance, and Road Infrastructure

The mode of transport to the hospital is a fundamental determinant in health seeking. This often presented a threat to the survival of women and newborns in developing nations. The common mode of transport in villages is by horse and donkey carts or at times bicycles. Most of these villages are in very remote rural areas with poor roads and far from regular routes for commuter taxis and/or private cars. The only available mode of transport is usually inconvenient for a woman in labour. Hence donkey carts were classified as too slow to address the immediate need of a woman in emergency. This is what a woman with haemorrhage had to say:

*“We left home late in the evening with a donkey cart in order to get to the main high way*…*this is the only means of transport we have. It was moving very slowly because the road condition was very bad*…*and*…*the place was getting dark*. *We reached the main high way after three hours*…*all this time I was bleeding with severe abdominal pains. Luckily*…*shortly after reaching the high way one private car gave us a lift to the hospital*…*but the baby was not born alive.” *

Even where horse carts are the common mode of transportation which is seemingly faster than donkeys, the roads may by be submerged in water, especially in the rainy season. The roads may also be so bumpy and paddy that the horse has to move very slowly so that the woman seeking health care might not endure severe pains that may worsen her condition;

*“It was during the rainy season and the roads were bad*…*like*…*paddy and full of stagnant water. I have to move the horse very slowly and carefully to avoid being stuck in the mud*…*this caused a lot of delay*…*but have to continue because I was worried and needed help. After a long and tiring journey...almost three hours*…*we arrived at the main road*…*but have *[*sic*]* to wait for another one hour before getting a commercial vehicle to the hospital”* (husband of 32-year-old woman with obstructed labour).

Even after surmounting a rough ride on a horse or donkey cart to get to the nearest health centre with a maternity, timely access to appropriate obstetric care is further hampered by inadequate logistics for evacuation of emergencies. Results from the interviews pointed out that there were instances when no ambulances were available for evacuation of patients because the one available had embarked on another referral. As a result, when a woman with an obstetric complication is referred to a second level, the family has to find alternative means of evacuation thus, costing more time and money. Others have to desperately resort to using the cart whatever the distance and time it may take which could lead to further complication. Narratives of husband of a 46-year-old woman with antepartum haemorrhage needing evacuation:

*“We used a horse cart to get to the nearest health centre*…*there I thought everything would be okay*…*but...that was not the case.*…*We were told to proceed to the next facility*…*but the ambulance was not there as it has taken another patient to the hospital*…*I have no option but to continue the journey with my horse*…*it was dark and has *[*sic*]* no money to pay for a taxi*…*after four hours we arrived at the second health facility*…*there she was examined and put in an ambulance to Bansang hospital*…*unfortunately, the baby did not survive.” *

Access problems still confronted women suffering from obstetric complications even if an ambulance was available at the first referral point en route to the main rural hospital. As many of the peripheral health centres in the study are on other side of the river, ambulances have to be ferried. However, this is usually hampered by high tides especially during the rainy season or mechanical problems with the ferry. It is not uncommon for rivers in the central and upper river regions in The Gambia to overflow their banks during mid rainy season hence rendering ferry service redundant. A 40-year-old woman explained her experience:

*“When we arrived at the river the ferry that was expected to take us to the other side was not there*…*some waiting passengers said it had a mechanical problem since morning*…*at that time I can't *[*sic*]* bear the pain any more*…*and the only means of crossing the river was to use a paddle canoe*…*and this caused a big delay.” *

Another woman who had haemorrhage explained her ordeal during evacuation when they reached a crossing point.

*“I started seeing blood in the afternoon after lunch*…*my husband took me to the main road with a donkey cart to look for transport to the health centre*…*we waited for a long time in vain as all the commercial vehicles in the area have *[*sic*]* already left for Basse*…*he then decided to continue the journey with the donkey to the nearest health centre. When we reached the health centre*…*I was examined by the nurse but she said they will refer me to Basse Health Centre [major health centre] 25km away. When we reached the crossing point the ferry was not working because the whole area was overflowed and*…*the water was moving very fast*…*we resorted to using a paddled canoe to get to the other bank where another ambulance was waiting.” *

## 5. Cost

The respondents explained how lack of money influences pregnant and labouring women's health seeking behaviour. Women attend antenatal care and if problems are observed they are advised to deliver at the hospital. However, the husbands and/partners may not have enough money to be able to pay for transport and/or emergencies as well as extra money for food while at the hospital. The only alternative is to reach out to other people in the village for help, which does contribute to delays in seeking health care. An 18-year-old woman with obstructed labour narrated:

*“My husband took me to the village health centre*…*but the nurses said the problem cannot be managed there*…*so they have to take me to the main hospital. At that time we don't *[*sic*]* have any money with us*…*my husband went back home*…*not very far*…*to negotiate for money while the ambulance was waiting. He got only D100.00 (equivalent US$3.8) as it was during night and most people were sleeping.” *

Another woman with obstructed labour had this to say:

“*…when I was in labour what prevented me from seeking health care early was the lack of money to hire a vehicle*…*we live in a very remote village and only managed to reach the high way on a donkey cart*…*I have to rely on my husband to make arrangements (*…*borrowing money from the nearby village) to pay for a car to drive us from the highway to the hospital.” *

In a related case, a husband of a 40-year-old woman with severe pre-eclampsia living 85 km away from the main hospital explained his frustrations in raising money for emergency services:

*“When the decision was reached to take her (his wife) to the hospital*…*I did not have any money on me...I had to borrow some money from a neighbour that night which was not enough*…*it took me two hours to raise this amount*…*and to beg the taxi driver to assist and accept the money in order to safe *[*sic*]* wife's life.” *

In some instances husbands were compelled to sell their assets to ensure that the woman gets to the hospital. A 20-year-old woman with severe pre-eclampsia/haemorrhage narrated:

*“He (her husband) gave us the little money he had on him*…*to raise more money he initially sold his goat and latter on his sheep*…*We are farmers so we barely have enough for the family upkeep not to mention about *[*sic*]* emergency funds”. *

A mother of a 26-year-old woman with antepartum haemorrhage also commented on the unavailability of money during emergencies:

*“…there used to be a community ambulance in this village that transport *[*sic*]* all women in labour to the hospital*…*but it had a breakdown at the time she (meaning her daughter) went into labour. The husband had no money at that time…and has *[*sic*]* to go out in the village to borrow money from shopkeepers in order to hire a bush taxi to get to the hospital*…*this was late in the night*…*” *

### 5.1. Blood Transfusion

Timely availability and access to life-saving interpositions such as blood transfusion are an essential component for the reduction of maternal and perinatal mortality. However, the hospital from which our patients were recruited did have a blood bank, but was not adequately stocked to meet the demands of the population. As a result, patients who require transfusions are compelled to obtain transfusion blood mostly from friends, relatives, and/voluntary donors, failure of which prolongs access to timely intervention. Scarcity of blood causes delays in receiving appropriate care even if family members are able to provide the required quantity, because of the time they need to go outside of the hospital in search of suitable donors. Thus, the timely delivery of appropriate emergency obstetric care services is further compromised. The following are testimonies from key informants during their stay at the hospital in their pursuit for potential blood donors:

*“*…*her blood was taken and sent to the machine (meaning laboratory)*…*latter on I was informed by the nurses that she lacks blood and will need blood transfusion*…*but*…*no blood was given. After waiting in vain*…*I went back to the nurses to enquire about the blood because my daughter-in-law was not improving*…*One nurse told me that the only thing to save her life is blood*…*so I should bring a blood donor as early as possible. I am old and can't donate blood*…*so I sent for her husband” *(mother-in-law of a 31-year-old anaemic woman).

A husband of a 20-year-old woman who had eclampsia and antepartum haemorrhage also had this to say:

*“After she (meaning his wife) delivered I was informed that she lacks blood*…*at that moment there was no blood in the hospital*…*I donated one bottle of blood but that was not enough*…*it was very difficult for me to get another suitable blood donor because I don't know anybody in this area*…*I latter sent a message for my younger brother from the village 8 km away who also came and donated one bottle of blood.” *

In rare circumstances women in critical conditions would be transfused a pint of blood on loan which the relatives have to replace. Getting a suitable donor is tiring and frustrating for the relatives as the people they usually bring are normally not suitable candidates, thus have to resort to the negotiating table with the nurses;

*“*…*the nurses told me that my wife lacked blood and needed to be transfused*…*I was then instructed to provide a blood donor*…*my son volunteered to donate but unfortunately he was not in the same blood group*…*after a long negotiation with the nurses*…*one bag of blood was provided and transfused on loan. *…* I have to go and look for donors to replace the blood*…*this is not an easy task for me” *(husband of a 30-year-old woman with antepartum haemorrhage).

A mother-in-law explained her encounter with the nurses at the hospital when the daughter-in-law with antepartum haemorrhage was found to be requiring blood;

*“*…*at the hospital we were told that her blood is very low so we should go and look for a blood donor*…*at that time her husband was not around and we are too old to donate*…*We begged them (nurses) to assist us safe *[*sic*]* her life by getting blood*…*latter on during the day one bottle of blood was provided and mounted on as a loan” *(mother-in-law of 29-year-old woman with APH).

## 6. Discussions

The current study applies the classic “three delay” model to explore delays of care in stillbirths and neonatal deaths. The majority of perinatal deaths that occurred in the current study were due to delay in reaching a health facility, transport/cost delays (delay 2). We also found household delays (delay 1) as the second important determinant of perinatal deaths. Most of the stillbirths were reported to be macerated, suggesting that the deaths probably occurred even before reaching the hospital. The first and second delays are significant because births still occur at home and access to appropriate care is still a major problem [15]. Thus, a call for strengthening community programmes seems warranted for improved newborn care in low-income countries [6], such as The Gambia. Our findings on the contribution of delays on perinatal deaths differ from studies conducted in Tanzania [15] and Uganda [16], where most newborn deaths were found to be associated with the first and third delays. 

Obstetric complications are similar around the world, but the risk of death is not. Ninety-nine of all maternal and perinatal deaths occur in developing countries [19]. Thus, women must have timely access to good quality obstetric care services at all times because complications are virtually inconceivable to anticipate and hard to forestall. In addition, families and/or communities must both realise and respect the available services. In our study, the inherent factors that contributed to stillbirths were similar to the classical list of the “three delays” in accessing EmOC [14]. However, in most cases these circumstances contribute first to the death of the child and later eventually also to the death of the mother. In stillbirths the women who survive and her relatives can tell their stories. The organisation of health care in poor countries is often fragmented based on subsided care packages. We emphasise that stillbirths share the same dilemmas as maternal deaths. Initiatives addressing mother and foetus as “one” and as such the pathways to solutions should be informed. The results of the current study illustrates that initiatives to make a change for maternal health would save many more lives than counted in a reduction of maternal mortality rate.

Medical care for women with obstetric complications begins with the recognition of danger signs [14]. Delays in recognizing danger signs, in seeking, reaching, and obtaining appropriate maternity care are key elements in maternal and perinatal deaths. However, recognition of the severity of a complication is an important determinant of the decision to seek help outside home. Pregnancy and childbirth are often considered as a natural process, thus signs and symptoms of complications are seldom recognized as reasons for concern. Thus, ineffective decision-making at the family level was identified as an important barrier in accessing timely EmOC in the present study. Similar findings have been reported elsewhere [20]. Birth preparedness and complication readiness (BP/CR), a strategy that promotes timely access and use of a skilled maternal and newborn care especially during childbirth, reduce delays in obtaining this care. Thus, birth preparedness motivates people to plan to have a skilled provider at every birth. If women and their families make the decision to seek health care before the onset of labour, and successfully follow through this plan, the woman will certainly reach and receive appropriate care before developing any potential complication during childbirth, thus averting the first two delays [21]. Even though women recognize danger signs, prompt decision to seek help outside of the home was not automatic as highlighted in the findings of the present study. Thus, it would be reasonable to speculate that failure in recognizing the severity of symptoms of obstetric complications could be one key reason for delay in seeking appropriate medical care in rural Gambia.

Women's liberty and status are also reported to have an influence in the utilization of obstetric services [22]. Low levels of female education and lack of empowerment and autonomy to make decisions deter women from seeking obstetric care in time. In the current study, women's choice to deliver at the hospital was heavily influenced by their husbands. It is not the pregnant woman herself who often decides on the place of birth but rather her family. In rural areas of The Gambia the husband and/or mothers-in-law are the most influential decision-makers. They make the final decision even if others offer their opinion. However, traditional birth attendants also played a crucial role in facilitating swift decision to seek health care. They tended to refer to their experiences with previous women in relation to pregnancy and childbirth in order to convince the family to seek health care. In resource-poor countries, particularly in rural settings with limited access to healthcare, traditional birth attendants are an essential source of obstetric care [23]. Thus, a programme geared towards educating families, women, and traditional birth attendants to recognize danger signs during pregnancy and childbirth as well as dangers associated with delay in seeking health care in the event of an obstetric complication seems warranted.

Timely referral during an obstetric emergency is a critical element in maternal and child health survival strategies. Once a decision is made that a complication needs medical intervention, availability of transport and easy accessibility to a facility with EmOC capabilities become crucial. In rural areas of The Gambia antenatal care is provided by outreach teams from peripheral health centres many of which don't have a maternity with appropriate surgical capacity. Thus, health facilities with appropriate obstetric capacity are usually at considerable distance from women who develop obstetric complications. In our study, traditional birth attendants reiterated that they received women who needed referral to the hospital, but delay in seeking and getting care was compounded by the lack of reliable transport and poor road network. The significant contribution of transport and mobility to development and the livelihood of people is widely recognized. Thus, appropriate, affordable, and timely transport in accessing health care should be recognized as a major developmental goal. Proponents argued that improved transportation and mobility directly improve access to health services and indirectly enhance aspects of social and economic development that impact positively on health. However, transportation to health facilities is not often seen as an essential element of access to care, but an individual challenge to overcome.

At the time of an obstetric emergency, every moment of delay in seeking and receiving skilled care increases the risk of maternal deaths/disability and stillbirths. However, unavailability and high cost of transportation, poor roads, and difficult river crossing and transportation from remote villages may increase the time to reach a hospital [24]. Long distance to the hospital and lack of appropriate means of transportation are major problems affecting the referral system in rural areas of The Gambia. During the rainy season most of the roads become difficult to navigate. For many remote rural dwellers in the Gambia, the uneven distribution of EmOC facilities means slow and long journeys that contribute to poor pregnancy outcomes and increased morbidity and mortality. Distance from health facilities and transport difficulties such as finding a motorised vehicle for patient transportation were found to be barriers in access of care [25]. Many preventable maternal and perinatal deaths continue to happen in remote rural settings in The Gambia. 

It has been documented that a functioning continuum of care between the home and the hospitals is needed to minimize pernicious delays and efficaciously connect pregnant women to skilled obstetric care [24]. Even though families in remote areas made a prompt decision to seek care poor, terrain and transportation difficulties posed unnecessary delays in reaching adequate care. Women trying to reach far-off facilities often fail, and those with life-threatening complications may suffer en route [14]. Evidence showed that a lack of mobility and appropriate transportation during an obstetric emergency contributes significantly to maternal and neonatal mortality in rural Gambia [26] and in Ghana, Nigeria, and Sierra Leon [14]. Earlier studies conducted in rural West African communities demonstrated transportation difficulties and distance from health facilities as major obstacles in accessing EmOC services [27]. Thus, it would be reasonable to presume that improved transportation during an obstetric emergency could directly ameliorate access to EmOC facilities that impact positively on maternal and neonatal health. We therefore believed that forging partnership between rural communities and local transport owners could be one key strategy in overcoming transport issues during emergency. This scheme was implemented in north west Nigeria and had led to significant diminution in transportation cost and maternal and perinatal mortality [28]. Though the availability of EmOC and motorised transport are vital for maternal and child survival during complications, women in remote settlements should be encouraged to move nearer to a health facility with a maternity as the delivery date approaches, and to stay there until the baby is born [29]; like in a maternal shelter or waiting home.

Cost is a key factor in determining the choice of place of delivery during an emergency, and it contributes to the first delay in seeking care outside home [20]. The cost of maternity care and the inability to meet the cost is a significant barrier to EmOC access, thus an important determinant of maternal and perinatal mortality [30]. Although maternity care is officially provided free of charge in public hospitals in The Gambia, indirect and/or opportunity costs still remain a substantial obstacle in timely access to obstetric care in rural settings. In their review article, Ensor and Cooper [25] considered distance and time as indirect and opportunity costs that influence uptake of health services in developing countries. Women's access to EmOC is further compromised if the care being sought is going to be very costly. The distance and time involved in seeking health care often determine how much cost a family may incur. Women seldom travel alone during an obstetric emergency; so accompanying family members increase the time lost in domestic work, cost of transportation and food during their stay in the hospital [22]. Thus, critical decisions are made to spend scarce resources in saving the life of women and babies. Poverty and high costs of health services has been reported by Essendi et al [20] as important barriers in the utilization of maternal health services among the poor. This is consistent with findings in the current study. Most families intended to access formal delivery services in time but, failed because they are poor and cannot afford the cost to pay for commercial vehicles at the time a decision was made during an emergency. In the Gambia, a majority of the rural poor lack stable and regular sources of income. As a result, they had to borrow money from shopkeepers and/or relatives to meet the cost of transportation and food while at the hospital. Others resorted to selling their farm products or animals. This is consistent with findings from studies conducted elsewhere in low-income countries [31].

In many developing countries the direct costs of transport constitute a significant proportion of the total health care expenditure. In northeast Brazil [32] the costs of accessing health facilities have been estimated to represent 25% of the total expenditure on health. The current study highlighted lack of money as an impediment in making prompt decision to seek care and in reaching a medical facility. This is corroborated by findings from a previous study in rural Gambia [26] and Bangladesh [33, 34]. The abolition of maternity fees could be part of the strategies to improve outcome and utilization of services, but this cannot be successful without complementary supply-side and increase investment. Like any health intervention, EmOC requires resources; personnel, time, equipment, drugs, and supplies; and resources are always limited [35].

Blood transfusion is one of eight key life-saving interventions that should be available in first-level referral health care facilities providing EmOC [36]. Although the need for blood is universal, millions of patients requiring transfusion do not have timely access to safe blood [37]. Shortage of blood and difficulty obtaining blood for transfusion are still a major obstacle in the management of obstetric emergencies in rural hospitals in The Gambia. This was previously reported by another author on a study on maternal mortality in rural Gambia [26]. The lack of access to and shortage of safe blood for women in this rural hospital could be explained by the increased demand for blood among women of reproductive age in the Gambia, many of whom are anaemic [38]. Additionally, many of the women in this study were admitted with severe bleeding which required immediate blood transfusion. This put a strain on the limited stock of blood in the bank. For blood to be readily available in hospitals, people must be willing to donate voluntarily. However, cultural beliefs (taboos) coupled with the fear of being tested positive for HIV during the screening of blood deters people from donating blood in rural Gambia. As a result potential donors seldom make themselves available for blood donation, thus creating a big gap between supply and demand. Timely, appropriate, and safe blood transfusion during pregnancy and childbirth can make a real difference between life and death for many women and their newborns [36], especially in rural settings.

Safe blood donors are the mainstay of safe and adequate supply of blood and blood products in hospitals. The safest blood donors are voluntary, nonremunerated blood donors from low-risk populations [39]. However, due to inadequate stock of blood supply, family/replacement or paid donors still provide a large quantity of blood collected in rural hospitals in The Gambia. As a result, timely access to transfusion service is jeopardised taking cognisant of the time the relatives have to spend in search for blood donors. It is important to note that the implication of enumerated blood donation increases the risk of transfusion transmitted infections, especially HIV and viral hepatitis [40]. Public health interventions to inform operating blood bank services seems warranted to reducing perinatal mortality rates in poor rural settings in the Gambia.

## 7. Methodological Considerations

The broad approach taking into consideration the influence of various individual, socio-cultural, and community factors on accessibility to EmOC services in rural Gambia is a strength of the current study. In addition, conducting interviews in homes as opposed to facility setting allows the respondents to speak their minds freely, thus minimising information bias. The advantage of this study design, using stillbirths to address the shortcomings, is that the women themselves can give information. This is not the case with maternal mortality. Finally, the short window period for the follow-up interviews may have also eliminated recall bias. 

Selection bias could play a role in the current study. A little more than half (52%) of the births in The Gambia take place in health facilities suggesting that a substantial proportion of women still give birth in home settings. Since the study aimed at exploring barriers to EmOC services in the catchment area of Bansang hospital, the findings may not be representative of the of the entire country with diverse geographic and sociodemographic differences. Thus, the results should be interpreted with caution. Systemic analysis of perinatal deaths in developing countries is hampered by a scarcity of data, especially in countries with nonexistent or incomplete vital registration systems, and where some events take place in homes and villages, not hospitals. However, the findings illustrate useful information regarding barriers to EmOC services for women trying to reach the hospital which is vital for reproductive health managers in The Gambia.

## 8. Conclusion

The barriers uncovered in this study are similar to the classical list of “three delays” in accessing life-saving emergency obstetric care interventions. Long distances to the hospital and lack of appropriate means of transportation were found to be major problems affecting timely evacuation of women with obstetric complications. Obtaining blood for transfusion was also identified as another impediment to the timely dispensation of obstetric care interventions. Maternal and perinatal deaths could be averted if all women delivered in a facility that has the capacity to provide EmOC and/or life-saving interventions, such as blood transfusion in the event of obstetric complications. Thus, improved and timely access to EmOC backed by emergency transportation seems warranted for improved maternal and neonatal survival in poor rural settings in The Gambia.

## 9. Key Issues/Observations

1. The constraints for maternal and perinatal health are similar. Care for the infant starts with proper pregnancy care for the mother including access to good-quality maternity care.
2. The “three delays” framework is a useful tool for addressing perinatal survival. These delays are addressed as individual problems, not part of an overall access to proper and good-quality public maternity care.
3. Access issues are important in perinatal survival in resource-poor areas of the world such as rural Gambia.

## Figures and Tables

**Table 1:** Types of delay experienced by women (*n* = 20).

Delays	*n* (%)
(1) First delays	
(i) Delay in recognising danger signs during pregnancy and labour	3 (15.0)
(ii) Sociocultural belief/family decision-making delays	3 (15.0)
(2) Second delays	
(i) Problems in transport, distance, and road infrastructure	5 (25.0)
(ii) Cost (no money)	5 (25.0)
(3) Third delays	
(i) Delays receiving blood transfusion	4 (20.0)
